# GPs’ mental wellbeing and psychological resources: a cross-sectional survey

**DOI:** 10.3399/bjgp17X691709

**Published:** 2017-07-18

**Authors:** Marylou Anna Murray, Chris Cardwell, Michael Donnelly

**Affiliations:** School of Medicine, Dentistry, and Biomedical Sciences, Centre for Public Health, Queen’s University Belfast, and Institute of Clinical Sciences, Royal Victoria Hospital, Belfast.; School of Medicine, Dentistry, and Biomedical Sciences, Centre for Public Health, Queen’s University Belfast, and Institute of Clinical Sciences, Royal Victoria Hospital, Belfast.; School of Medicine, Dentistry and Biomedical Sciences, Centre for Public Health, and UKCRC Centre of Excellence for Public Health, Queen’s University Belfast, and Institute of Clinical Sciences, Royal Victoria Hospital, Belfast, Northern Ireland, UK.

**Keywords:** optimism, primary care, psychological, resilience, surveys and questionnaires

## Abstract

**Background:**

The negative impact of work has been the traditional focus of GP surveys. We know little about GP positive mental health and psychological resources.

**Aim:**

To profile and contextualise GP positive mental health and personal psychological resources.

**Design and setting:**

Cross-sectional survey of GPs working in Northern Ireland (NI).

**Method:**

A questionnaire comprising the Warwick Edinburgh Mental Wellbeing Scale (WEMWBS) and measures of resilience, optimism, self-efficacy, and hope, and sociodemographic information was posted to 400 GPs randomly selected from a publicly available GP register.

**Results:**

The response rate was 55% (n = 221 out of 400). Mean value for GP wellbeing (WEMWBS) was 50.2 (standard deviation [SD] 8) compared to UK vets 48.8 (SD 9), UK teachers 47.2 (SD 9), and the population of NI 50.8 (SD 9). After adjustment for confounding, mean WEMWBS was 2.4 units (95% CI = 0.02 to 4.7) higher in female GPs than males (*P* = 0.05), and 4.0 units (95% CI = 0.8 to 7.3) higher in GPs ≥55 years than GPs ≤44 years (*P* = 0.02). Optimism was 1.1 units higher in female GPs than male GPs (95% CI = 0.1 to 2.0), and 1.56 units higher in GPs ≥55 years (95% CI = 0.2 to 2.9) than in those ≤44 years. Hope was 3 units higher in GPs ≥55 years (95% CI = 0.4 to 5.7) than in those aged 45–54 years. Correlation between WEMWBS and psychological resources was highest with hope (*r* = 0.65, *P* < 0.001).

**Conclusion:**

GPs have levels of positive mental health that are comparable to the local population and better than other occupational groups, such as vets and teachers. Male and younger GPs may have most to gain from wellbeing interventions.

## INTRODUCTION

Increasing sub-specialism within secondary care and ongoing demographic shifts highlight the pivotal role that GPs are required to play in the provision of personalised patient care. In the context of rising GP workload and workforce concerns, there is a need to explore the positive resources and strengths of GPs. This positive approach complements the traditional focus on illness, stress, depression, and burnout, as well as recognising GP resilience as a resource in the context of work pressures. Levels of positive mental health vary across populations and occupational groups. The definition and measurement of resilience continues to be a source of debate,^1^^,^^2^ and empirical evidence for resilience training programmes is limited.^3^ GPs have expressed ethical-based concerns regarding the recommendation that they should undertake resilience training to adapt to increasingly difficult working conditions.^4^^–^6 This article examines these arguments by measuring GP wellbeing, including resilience and three related psychological resources that are amenable to change and known to impact on work performance.^7^ More specifically, the authors assess the profile of positive mental health and level of personal psychological resources among GPs, including the nature and degree of variation in GP positive mental health and psychological resources in terms of age, sex, GP practice size, and rurality. The relationships are then explored between GP positive mental health and their personal psychological resources.

## METHOD

Participants were identified from a publicly available register of GPs compiled by the Business Services Organisation (BSO) in Northern Ireland. The list includes principals and salaried GPs (*n* = 1267), but not training or locum GPs. A random sample of 400 GPs was drawn from this list using a random number generator,^8^ and in expectation of 50% attrition. A personalised invitation, consent form, information sheet, stamped response-indicating postcard, stamped-return envelope, and a questionnaire were mailed to GPs in January 2016. A reminder with replacement questionnaire was mailed in February 2016 to GPs who had not returned a signed, response-indicating postcard. The questionnaire comprised five instruments with good psychometric properties, international validation, and brevity, mindful of respondent burden. The Warwick-Edinburgh Mental Wellbeing Scale (WEMWBS) has been validated in general populations^9^^–^11 and used to measure positive mental health in occupational groups.^12^^,^^13^ GP personal psychological resources were assessed using the Brief Resilience Scale (BRS),^1^^,^^14^ the Life-Orientation Test (LOT-R) of Optimism,^15^^,^^16^ the General Self Efficacy (GSE) scale^17^ (which addresses the perceived ability to cope with daily hassles and adapt to stressful events), and the Adult State Hope Scale (ASHS).^18^ The BRS was chosen because it assesses resilience in terms of being a malleable and modifiable personal resource.^19^ Information was collected on key sociodemographic variables, including broad age categories, sex, practice size, and location.

**How this fits in**

This survey presents novel insights into the positive resources and strengths of GPs complementing the traditional focus on burnout and stress. In a context of substantial flux, GPs are maintaining levels of positive mental health comparable to the local population. They appear to have good levels of psychological resources, particularly with respect to adopting an optimistic and hopeful attitude to life and work.

### Study size calculation

A standard deviation (SD) of 9 20^,^^21^ was used to calculate sample size of 200 based upon determining the true mean WEMWB score in GPs to within +1.2 or −1.2 units. A sample of 200 respondents afforded the detection of a potential difference of 3.75 units in WEMWB scores (with 80% power at the 5% level) between GPs with higher than median practice size compared with lower than median practice size. It also allowed the detection of a true correlation of 0.2 between personal psychological resources (as measured by BRS, AHS, GSE, and LOT- R) and positive mental health or WEMWBS (with 80% power at the 5% level).

### Data analysis

The questionnaire data were collected in paper format and entered into SPSS Version 21.0 for statistical analysis. All scales approximated to a normal distribution. Means and SDs were calculated for the components and overall scores of the WEMWB Scale and the four measures of personal psychological resources (BRS, ASHS, LOT-R, and GSE scores) (Table 1). Independent samples *t*-tests were used to compare mean scores by sex (male/female), location (urban/rural), and practice size (≤4 GPs/≥5 GPs). Analysis of variance (ANOVA) was used to compare WEMWB scale by age category (≤44, 45–54, ≥55 years). Linear regression was used to analyse the increase in WEMWB scale per unit increase in age category, and to test for trend. It was also used to determine difference in mean WEMWB scores by categorical variable, adjusting for potential confounding by age, sex, location, and practice size. R^2^ statistics were calculated for adjusted models. Pearson correlation coefficients (and accompanying *P*-values) assessed the association between WEMWBS and the four resource scales. Individual missing items within scales were uncommon. Where an item was missing the score for the entire scale was omitted from the analysis. Means and standard deviations were extracted from relevant studies that used each of the five instruments in order to set the results in a comparative international context. The studies were obtained with the help of a specialist librarian using a systematic search of Medline, PsycInfo, and Embase databases (further details on search strategy and results are available from the authors).

**Table 1.** Profile of GP positive mental health and psychological resources

	**Positive mental health (WEMWBS), scale range 14–70**			**Optimism (LOT–R), scale range 0–24**			**Resilience (BRS), scale range 1–5**			**Self-efficacy (GSE), scale range 10–40**			**Hope (ASHS), scale range 6–48**		
				
	***n***	**Mean (SD)**	***P***	***n***	**Mean (SD)**	***P***	***n***	**Mean (SD)**	***P***	***n***	**Mean (SD)**	***P***	***n***	**Mean (SD)**	***P***
**Total**	**214**	**50.2 (8)**		**215**	**15.6 (3.3)**		**221**	**3.35 (0.7)**		**213**	**30.3 (3.8)**		**212**	**34.5 (6.7)**	

**Sex**															
Male	111	49.6 (7.6)	0.12	109	15.3 (3.4)	0.06	112	3.3 (0.6)	0.57	109	30.6 (4)	0.37	109	34.4 (6.7)	0.56
Female	92	51.4 (8.4)		90	16.2 (3.2)		92	3.4 (0.7)		88	30.1 (3.5)		87	35.0 (6.2)	

**Location**															
Urban	112	50.0 (7.5)	0.60	110	15.4 (3.2)	0.18	113	3.3 (0.7)	0.27	111	29.9 (3.6)	0.12	110	34.7 (6.5)	0.57
Rural	85	50.6 (8.6)		83	16.0 (3.5)		85	3.4 (0.6)		80	30.7 (3.8)		80	34.2 (6.8)	

**Partners**															
≤4 GPs	114	50.0 (7.7)	0.52	113	15.8 (3.4)	0.58	115	3.4 (0.6)	0.31	109	30.2 (3.5)	0.81	109	34.2 (6.8)	0.53
≥5 GPs	91	50.7 (8.4)		89	15.5 (3.2)		92	3.3 (0.7)		91	30.4 (4.2)		90	34.8 (6.6)	

**Age**															
≤44	86	49.7 (7.8)		82	15.5 (3.2)		86	3.4 (0.6)		86	29.9 (3.1)		85	34.5 (5.7)	
45–54	79	49.6 (7.9)	0.21^a^	79	15.4 (3.4)	0.42^a^	79	3.3 (0.7)	0.36^a^	75	30.5 (4.2)	0.57^a^	75	33.3 (7.4)	0.14^a^
≥55	45	52.1 (8.2)		45	16.1 (3.1)		47	3.4 (0.6)		44	30.4 (4.3)		44	35.7 (6.7)	

a P-value from ANOVA, analysis of variance. ASHS = Adult State Hope Scale. BRS = Brief Resilience Scale. GSE = General Self Efficacy Scale. LOT-R = Life Orientation Test of Optimism. SD=standard deviation. WEMWBS = Warwick Edinburgh Mental Wellbeing Scale. See Appendices 1 and 2 for details of missing data across sociodemographic and outcome variables.

## RESULTS

### Participants

The response rate was 55% and the characteristics of respondents (*n* = 221 out of 400) were comparable to the study sample and GP population profile, with the exception of a higher than expected number of rural GPs who responded (Appendix 1). The Brief Resilience Scale was completed by all respondents (see Appendices 1 and 2 for details of missing outcome and demographic data across variables). The population profile of positive mental health and psychological resources is presented in Table 1. Mean values for each construct were in the top quartile of the scale range. In crude analyses (that is, not adjusted for confounders), female GPs had higher mean values for positive mental health and for each psychological resource (except self-efficacy) than men, although these differences were not significant. Similarly, rural GPs had higher scores than urban GPs across measures. However, in these crude analyses the authors did not find any statistically significant differences in wellbeing and psychological resources between groups based upon rurality and number of partners.

After adjustment for confounding, female GPs and older GPs had statistically significant higher mean WEMWB scores (Table 2). Mean WEMWB scores were 2.4 units higher in females than males (95% CI = 0.02 to 4.7), and 4.0 units higher in GPs ≥55 years than in GPs ≤44 years (95% CI = 0.8 to 7.3). Similar statistically significant findings were observed for optimism (data available from authors). The adjusted mean optimism score was 1.1 units higher in females than males (95% CI = 0.1 to 2.0), and 1.56 units higher in GPs >55 years compared to those ≤44 years (95% CI = 0.2 to 2.9). In the adjusted model, hope scores were 3 units higher in GPs ≥55 years than those in the 45–54 age group (95% CI = 0.4 to 5.7). Statistical analyses did not indicate differences between measured GP characteristics and self-efficacy or resilience scores. (Further details of adjusted analyses are available from the authors).

**Table 2.** GP positive mental health (WEMWBS) scores across demographic and practice variables

	***n***	**Mean (SD)**	***P***	**Difference in mean (95% CI)**	***P* for trend**	**Adjusted^a^ difference in mean (95% CI)**	***P* for trend**
**Total**	**214**	**50.2 (8)**					

**Sex**							
Male	111	49.6 (7.6)	0.1	−1.7 (−3.9 to 0.5)	0.12	−2.4 (−4.7 to −0.02)	0.05
Female	92	51.4 (8.4)		0 (ref cat)		0 (ref cat)	

**Location**							
Urban	112	50.0 (7.5)	0.6	−0.6 (−2.9 to 1.7)	0.6	−0.8 (−3.1 to 1.5)	0.5
Rural	85	50.6 (8.6)		0 (ref cat)		0 (ref cat)	

**Number of partners**							
≤4	114	50 0 (7.7)	0.5	−0.7 (−2.9 to 1.5)	0.5	−1.1 (−3.5 to 1.2)	0.3
≥5	91	50.7 (8.4)		0 (ref cat.)		0 (ref cat)	

**Age**							
≤44	86	49.7 (7.8)		−2.3 (−5.2 to 0.5)	0.11	−4.0 (−7.3 to −0.8)	0.02
45–54	79	49.6 (7.9)	0.2^b^	−2.4 (−5.3 to 0.5)	0.1	−3.5 (−6.8 to −0.3)	0.03
≥55	45	52.1 (8.2)		0.0 (ref cat)	(0.16)	0.0 (ref cat)	(0.03)

a Model contains sex, urban/rural, number of GPs, and age. R^2^ = 0.12.

b P-value from ANOVA, analysis of variance. ref cat = reference category. WEMWBS = Warwick Edinburgh Mental Wellbeing Scale. Missing data across demographic variables: See Appendices 1 and 2 for details of missing data across sociodemographic and outcome variables.

Table 3 shows that each scale or measure of resource was associated positively, albeit moderately so, with mental wellbeing. The strongest correlation was with hope (*r* = 0.65), and the weakest was with self-efficacy (*r* = 0.35). Overall, hope had the strongest relationships with the set of wellbeing and resource variables, including self-efficacy (*r* = 0.48).

**Table 3.** Correlation coefficients^a^ between positive mental health and psychological resources

**Variables**	**Optimism (LOT-R)**	**Resilience (BRS)**	**Self-efficacy (GSE)**	**Hope (ASHS)**
**WEMWBS**	0.55	0.50	0.35	0.65
**LOT-R**	^*^	0.57	0.32	0.59
**BRS**	^*^	^*^	0.51	0.45
**GSE**	^*^	^*^	^*^	0.48
**ASHS**	^*^	^*^	^*^	^*^

a All correlations were significant (2-tailed) at <0.001. ASHS = Adult State Hope Scale. BRS = Brief Resilience Scale. GSE = General Self Efficacy Scale. LOT-R = Life Orientation Test of Optimism. WEMWBS = Warwick Edinburgh Mental Wellbeing Scale.

## DISCUSSION

### Summary

To the authors’ knowledge this study presents for the first time the positive mental health or wellbeing of GPs and their level of personal psychological resources. Overall, the positive mental health of GPs was at least comparable to the local population and better than other occupational groups, such as vets and teachers. GPs in the oldest age band (≥55 years) had the highest level of positive mental health, hope, and optimism, and female GPs had higher positive mental health and optimism than their male colleagues. GPs appear to have good levels of psychological resources, particularly with respect to self-efficacy and adopting an optimistic attitude to life and work. A hopeful attitude was the psychological resource that was most strongly related to positive mental health, followed by an optimistic outlook.

### Strengths and limitations

The response rate was relatively low at 55% and the wellbeing and psychological resource levels of non-respondents were unknown. It is possible that levels may be lower or higher than the authors have found, for example, respondents may be more (or less) optimistic. However, the response rate was comparable to published GP surveys,^22^ the sample of respondents reflected the GP population from which the study sample was selected randomly, and the sample size had adequate power to be confident about statistical estimates. It was necessary to use wide age bands in order to ensure anonymity. However, anonymity is likely to have moderated potential for social desirability bias.^23^ Correlation coefficients between positive mental health and measures of psychological resource may have been affected by common method variance.^24^ The cross-sectional design precluded causal inferences. It might be argued that biases are inherent in self-report measures, but there is good psychometric evidence for each measure. Also, the validated instruments have made significant contributions to their respective literatures and afford an opportunity for the survey findings to be interpreted in an international context.

### Comparison with existing literature

Although the level of positive mental health among the GP sample was lower than reported in previously published UK general population surveys,^9^^,^^10^ it was comparable to the level observed in a recent Northern Irish population survey,^11^ and higher than levels reported for other occupational groups in the UK, such as university employees,^25^ vets,^26^ and teachers.^13^ This new insight into GP mental health suggests that there may be merit in extending the focus of GP wellbeing beyond negative constructs such as burnout and stress. Similar to general population survey findings, older GPs had the highest level of positive mental health,^10^^,^^20^ perhaps reflecting a ‘stage of life’ sense of comfortable competency and achievement, and perceived positive impact of anticipated retirement. It is interesting to contrast the relatively high level of positive mental health in older GPs, with reports that the proportion of GPs aged 55–64 years who left practice doubled in the period between 2005 and 2014.^27^ This finding of higher wellbeing in female GPs contrasts with the results of general population surveys,^9^^,^^11^^,^^28^^,^^29^ and diverges from recognised sex differences in the prevalence of depression.^30^ Higher positive mental wellbeing scores in female GPs may be explained in terms of variables that were not assessed in this study, such as different work-time patterns between females and male GPs. For example, role conflict and work–family balance influence wellbeing,^31^ and part-time compared to fulltime work is associated with higher life satisfaction among career women.^32^ While the relationship between burnout and hours worked is dependent on the extent to which work arrangements meet the needs of doctors, their partners, and children.^33^

Interestingly, females and older GPs were more optimistic in their outlook than other GPs. U-shaped age variation in optimism has been described in three UK population samples.^34^ Perhaps unsurprisingly, context and circumstances appear to play a role. For example, one international study found that young, highly educated, affluent Irish females were the most optimistic.^35^ This GP sample was more optimistic than general population samples in the UK,^34^ Portugal,^36^ and Germany,^37^ although a higher level of optimism was reported by other occupational groups, such as nurses^38^ and military personnel.^39^ Optimism appears to be a significant predictor of physical health outcomes,^40^^–^42 including decreased mortality,^43^ as well as being related to better subjective wellbeing in times of adversity, and higher levels of engagement, coping, and being proactive in personal health protection.^44^ The authors’ finding of a moderately positive association between resilience and optimism concurs with studies of resilience in healthcare.^45^^,^^46^ Positive attitudes including optimism, tolerance, and humour, and celebrating small gains have been identified as pertinent to resilience in GPs.^47^^–^49 ‘Learned optimism’ forms the basis of the Penn Resilience programme,^50^ highlighting the potential to exploit synergism between these psychological resources. Furthermore, physician resilience has been defined as the

*‘... ability to invest personal resources in a way that initiates positive resource spirals despite stressful work conditions’*.^49^

The finding of a moderately strong correlation between resilience and positive mental health resonates with the concept of a resource spiral and is consistent with evidence that positive emotions promote positive adaptation to adversity.^51^ Upward spirals generated by positive emotions increase mental flexibility, a commonly identified attribute of resilient individuals.^45^^,^^52^^–^55 Normative Brief Resilience Scale scores were higher for health care professionals than this sample, and may reflect depleted resources in the face of increasing pressures in general practice. GPs’ perceptions about their level of general self-efficacy was comparable to other occupational groups, including health care professionals,^38^^,^^56^^–^58 and higher than general population samples.^59^^–^61

Perceived general self-efficacy appears to moderate the effect of daily hassles on positive wellbeing and negative mental health, and is a predictor of positive mental health.^61^^,^^62^ GPs’ relatively higher levels of self-efficacy may point to ways in which to support the GP workforce, as self-efficacy and job satisfaction are positively related.^63^

Hope, too, correlates positively with job satisfaction and work happiness, and is negatively associated with job stress.^63^^,^^64^ Unsurprisingly, hope had the strongest relationship with positive mental health in this study. The authors’ finding of a high correlation between hope and optimism has been identified in clinical and occupational groups.^64^^,^^65^ Collectively, the results add to evidence about the relationships between psychological resources such as hope and resilience and health and wellbeing in a work context.^66^^–^68 Also, it is likely that this resources-positive mental health-context set of relationships is influenced by other variables, such as organisational factors and social networks. A composite construct comprised of hope, optimism, resilience, and self-efficacy is associated with higher job satisfaction, less burnout, and lower work-related stress in doctors.^69^^–^71 Brief and web-based interventions have been shown to develop resources within this composite construct.^72^^,^^73^

### Implications for research and practice

These findings suggest that younger male GPs may experience lower levels of positive mental health than their older female colleagues, and that they might benefit from support measures designed to improve their wellbeing, such as coaching,^74^^–^76 or mindfulness.^77^^–^80 The relatively high levels of optimism, hope, and positive mental health in older GPs may have implications for morale and recruitment. Since evidence for resilience training (including programme content and format) is limited, a composite approach designed to capitalise on the synergism between related psychological resources simultaneously warrants further investigation.

## Appendix 1. Sociodemographic characteristics

**Table:**

**Characteristic**	**Respondents (*n* = 221)**	**Study sample (*n* = 400)**	**NI GP population (*n* = 1267)**
**Sex, *n*(%)**			
Male	112 (51)	201 (50)	645^a^ (51)
Female	92 (42)	199 (50)	622^a^ (49)
Missing	17 (7)		

**Age, *n*(%)**			
≤44	86 (39)	159^b^ (40)	
45–54	79 (36)	167^b^ (42)	
≥55	47 (21)	74^b^ (18)	
Missing	9 (4)		

**Location, *n*(%)**			
Urban	113 (51)	323 (81)	1043 (82)
Rural	85 (38)	77 (19)	224 (18)
Missing	23 (11)		

**Practice size, *n*(%)**			
≤4 partners	115 (52)	199 (50)	631 (50)
≥5 partners	92 (42)	201 (50)	636 (50)
Missing	14 (6)		

a Business Services Organisation (BSO) data states 50:50 male:female. BSO list does not provide specific gender information or forenames for all entries. Forenames were available for 622 females.

b Approximate age groups based on years registered on NI GP Performer’s List: ≤17, 18–30 and ≥31 years on Performer’s List.

## Appendix 2. Missing data for outcome variables and associated demographic variables

**Table:**

	**Incomplete or omitted scales, *n***	**Sex, *n***	**Practice location, *n***	**Practice size *n***	**Age,**
**Positive mental health, WEMWBS**	7	11	17	9	4
**Optimism, LOT-R**	6	16	22	13	9
**Resilience, BRS**	0	17	23	14	9
**Self-efficacy, GSE**	8	16	22	13	8
**Hope, ASHS**	9	16	22	13	8

ASHS = Adult State Hope Scale. BRS = Brief Resilience Scale. GSE = General Self Efficacy Scale. LOT-R = Life Orientation Test of Optimism. WEMWBS = Warwick Edinburgh Mental Wellbeing Scale.
